# Persistent Lyme Empiric Antibiotic Study Europe (PLEASE) - design of a randomized controlled trial of prolonged antibiotic treatment in patients with persistent symptoms attributed to Lyme borreliosis

**DOI:** 10.1186/s12879-014-0543-y

**Published:** 2014-10-16

**Authors:** Anneleen Berende, Hadewych JM ter Hofstede, A Rogier T Donders, Henriët van Middendorp, Roy PC Kessels, Eddy MM Adang, Fidel J Vos, Andrea WM Evers, Bart Jan Kullberg

**Affiliations:** Department of Internal Medicine, Division of Infectious Diseases, Radboud University Medical Center, Nijmegen, 6500 HB The Netherlands; Department for Health Evidence, Radboud University Medical Center, Nijmegen, 6500 HB The Netherlands; Institute of Psychology, Health, Medical, and Neuropsychology Unit, Leiden University, Leiden, 2300 RB The Netherlands; Department of Medical Psychology, Radboud University Medical Center, Nijmegen, 6500 HB The Netherlands; Department of Neuropsychology, Radboud University Medical Center, Nijmegen, 6500 HB The Netherlands; Donders Institute for Brain, Cognition and Behaviour, Radboud University Nijmegen, Nijmegen, 6500 HE The Netherlands; Department of Internal Medicine, Sint Maartenskliniek, Nijmegen, 6500 GM The Netherlands

**Keywords:** Lyme disease, Borreliosis, Persistent symptoms, Study protocol, Treatment, Doxycycline, Clarithromycin, Hydroxychloroquine, Placebo, Guidelines

## Abstract

**Background:**

Lyme borreliosis, a potentially severe tick-borne infection caused by *Borrelia burgdorferi*, can cause multi-system inflammatory disease. The incidence has been increasing, as has the number of patients with persistent symptoms attributed to *Borrelia*. These symptoms, also referred to as post-Lyme disease syndrome, may follow an erythema migrans or other Lyme manifestations, and include pain, fatigue, and cognitive disturbances. The optimal duration of treatment for these symptoms is a subject of controversy. The PLEASE study is designed to determine whether prolonged antibiotic treatment leads to better patient outcome than standard treatment.

**Methods/Design:**

The PLEASE study is a double-blind, randomized, placebo-controlled trial. Based on power analysis and compensating for possible loss to follow-up, a minimum of 255 patients with borreliosis-attributed persistent symptoms are included. These symptoms are either (a) temporally related to an erythema migrans or otherwise proven symptomatic borreliosis, or (b) accompanied by a positive *B. burgdorferi* IgG or IgM immunoblot. All patients receive open-label ceftriaxone for two weeks. Patients are then randomized (ratio 1:1:1) to blinded oral follow-up treatment for 12 weeks with (I) doxycycline, (II) clarithromycin combined with hydroxychloroquine, or (III) placebo. The primary outcome is the physical component summary score (PCS) of the RAND-36 Health Status Inventory (RAND SF-36) at week 14. Secondary outcomes include physical and mental aspects of health-related quality of life (assessed by the subscales of the RAND SF-36), fatigue, neuropsychological evaluation, physical activity, and cost-effectiveness.

**Discussion:**

This article describes the background and design issues of the PLEASE study protocol. The results of this study may provide evidence for prescribing or withholding prolonged antibiotic treatment.

**Trial registration:**

ClinicalTrials.gov: NCT01207739, Netherlands Trial Register: NTR2469

**Electronic supplementary material:**

The online version of this article (doi:10.1186/s12879-014-0543-y) contains supplementary material, which is available to authorized users.

## Background

Lyme borreliosis, the most common tick-borne infection in America, Europe, and Northern Asia, is a multi-system inflammatory disease caused by the spirochete *Borrelia burgdorferi sensu lato*. During the past two decades, the incidence has been increasing. In the USA, the number of reported borreliosis cases has doubled from 9,908 cases in 1992 to 19,931 in 2006 [1]. Incidence rates vary per state but have increased substantially over the last decade, with an incidence up to 75 cases per 100,000 persons in 2012 [2]. In the Netherlands, the incidence of the most common form of borreliosis, erythema migrans (EM), has risen from 39 per 100,000 in 1994 to 134 per 100,000 persons in 2009 [3]-[5].

In parallel with the growing incidence of early Lyme disease, the number of patients with persistent symptoms attributed to infection with *B. burgdorferi* seems to increase as well. These borreliosis-attributed persistent symptoms, also referred to as post-Lyme disease syndrome, chronic Lyme disease, or (true or presumed) persistent Lyme disease, may follow an EM or other, possibly unnoticed, manifestations of early Lyme disease, regardless of initial appropriate antibiotic treatment. Patients mainly present with pain, fatigue, neurological, and cognitive disturbances [6]-[8]. Three months after treatment of an EM, the prevalence of these symptoms can be as high as 25% [9]. Although this percentage tends to decrease as more time elapses, symptoms are often disabling, and influence the daily life of these patients. Especially chronic pain has been shown to be an important contributor to impairment of health-related quality of life, and is similar to that reported by patients with osteoarthritis [10].

So far, no general, well-accepted definition of the syndrome of borreliosis-associated persistent symptoms exists [11]. This has resulted in a lack of data on its incidence and prevalence, and has contributed to confusion and controversy. This controversy especially relates to the pathogenesis of borreliosis-attributed persistent symptoms: whether they emerge from an ongoing infection, are a post-infectious problem, or are not related to a *Borrelia* infection at all. Currently available diagnostic tools (primarily based on serology) are appropriate for the diagnosis of early Lyme disease in most cases, but have little value for the diagnosis of potentially persistent *Borrelia* infection [12]. As IgG antibodies against *Borrelia* may persist for many months or even years after acute infection, positive serology is not an indicator of active or persistent *Borrelia* infection [13],[14]. As long as there is no specific laboratory test for active infection, the decision whether and how long patients with persistent symptoms should be treated depends on evidence from clinical studies. However, as this evidence has not been consistent, two different approaches exist for patients with borreliosis-attributed persistent symptoms: (1) standard short-term treatment for 2-4 weeks, as advised for most manifestations of Lyme borreliosis by the Infectious Diseases Society of America (IDSA) [15] or (2) long-term treatment for at least 3 months, as advised by the International Lyme and Associated Diseases Society (ILADS) [16]. Previous randomized clinical trials have not convincingly demonstrated beneficial effects of prolonged antibiotic treatment [10],[17],[18], and have been subject of ongoing debate [19].

To obtain more insight into the optimal treatment regimen for patients with borreliosis-attributed persistent symptoms, we designed a double-blind, randomized clinical trial to compare short- versus long-term treatment. In this 3-arm study, entitled Persistent Lyme Empiric Antibiotic Study Europe (PLEASE), ceftriaxone followed by doxycycline (arm 1) or ceftriaxone followed by the combination of clarithromycin and hydroxychloroquine (arm 2) are compared to short-term therapy with ceftriaxone followed by placebo (arm 3). Here, we describe the study protocol.

## Methods/Design

### Study design

A randomized, double-blind, placebo-controlled trial is performed to determine whether long-term antibiotic treatment (ceftriaxone followed by doxycycline or ceftriaxone followed by the combination of clarithromycin and hydroxychloroquine) leads to better patient outcome than short-term treatment (ceftriaxone followed by placebo) in patients with borreliosis-attributed persistent symptoms. This prospective 3-arm study is conducted at two sites in the Netherlands, the Radboud university medical center (Radboudumc) and the Sint Maartenskliniek, and has been approved by the Medical Ethics Review Committee CMO Regio Arnhem-Nijmegen (registration number 2009/187, NL27344.091.09). The study is conducted in accordance with the principles stated in the most recent version of the Declaration of Helsinki and the International Conference on Harmonisation (ICH) guidelines on Good Clinical Practice.

### Study population

All patients are recruited from the outpatient clinic of the Radboudumc, after nationwide referral by physicians. The Radboudumc serves as one of the tertiary referral centers for the Netherlands' population of around 17 million. Screening is done using standard clinical and laboratory protocols. Eligibility is assessed by a physician according to specific inclusion and exclusion criteria (Table 1). In short, patients with borreliosis-attributed persistent symptoms (musculoskeletal pain, arthritis, arthralgia, neuralgia, sensory disturbances, or neuropsychological/cognitive disorders, with or without persistent fatigue) are eligible if these symptoms are either (a) temporally related to an erythema migrans or otherwise proven symptomatic borreliosis, or (b) accompanied by a positive *B. burgdorferi* IgG or IgM immunoblot. An eligible patient is asked to sign informed consent after obtaining written information about the study.

**Table 1:** **Inclusion and exclusion criteria**

Inclusion criteria	
1	Males or non-pregnant, non-lactating females who are 18 years or older
2	Complaints of musculoskeletal pain, arthritis, arthralgia, neuralgia, sensory disturbances (such as paraesthesias or dysesthesias), or neuropsychological/cognitive disorders, with or without persistent fatigue, that are:
A	either temporally related to an episode of erythema migrans or otherwise proven symptomatic Lyme borreliosis (defined as within 4 months after erythema migrans as assessed by a physician, or positive biopsy, PCR, culture, or intrathecal *B. burgdorferi* antibodies)
B	or accompanied by a positive *B. burgdorferi* IgG or IgM immunoblot (as defined by strict criteria in line with the European Union Concerted Action on Lyme Borreliosis (EUCALB) and the manufacturer of the immunoblot* [20],[21]), regardless of prior ELISA IgG/IgM screening results
3	Subjects must sign a written informed consent form
**Exclusion criteria**	
1	Subjects with a known history of allergy or intolerance to tetracyclines, macrolides, hydroxychloroquine, or ceftriaxone
2	Subjects who have had more than 5 days of antimicrobial therapy with activity against *B. burgdorferi* within the previous 4 weeks
3	Subjects with a presumed diagnosis of neuroborreliosis (CSF pleiocytosis or intrathecal antibody production) for which intravenous antimicrobial therapy is required
4	Subjects with a known diagnosis of HIV-seropositivity or other immune disorders
5	Subjects with positive syphilis serology or signs of other spirochetal diseases
6	Subjects with moderate or severe liver disease defined as ALP, ALT, or AST greater than 3 times upper limit of normal
7	Subjects who are receiving and cannot discontinue cisapride, astemizole, terfenadine, barbiturates, phenytoin, or carbamazepine
8	Subjects who are currently enrolled on other investigational drug trials or receiving investigational agents
9	Subjects who have been previously randomized into this study
10	Severe physical or psychiatric co-morbidity that interferes with participation in the study protocol, including previous medical diagnosis of rheumatic conditions, chronic fatigue syndrome, or chronic pain conditions, as well as insufficient command of the Dutch language
11	Co-morbidity that could (partially) account for the symptoms of the subject (e.g., vitamin B12 deficiency, anemia, hypothyroidism)
12	Subjects of child-bearing potential unwilling to use contraception methods other than oral contraceptives during the study therapy period

Abbreviations: PCR = polymerase chain reaction, CSF = cerebrospinal fluid, ALP = alkaline phosfatase, ALT = alanine aminotransferase, AST = aspartate aminotransferase.

*EUROLINE-WB: Anti-Borrelia (whole antigen plus recombinant VlsE). EUROIMMUN Corporation, Lübeck, Germany.

### Randomization and blinding

**Figure 1:**
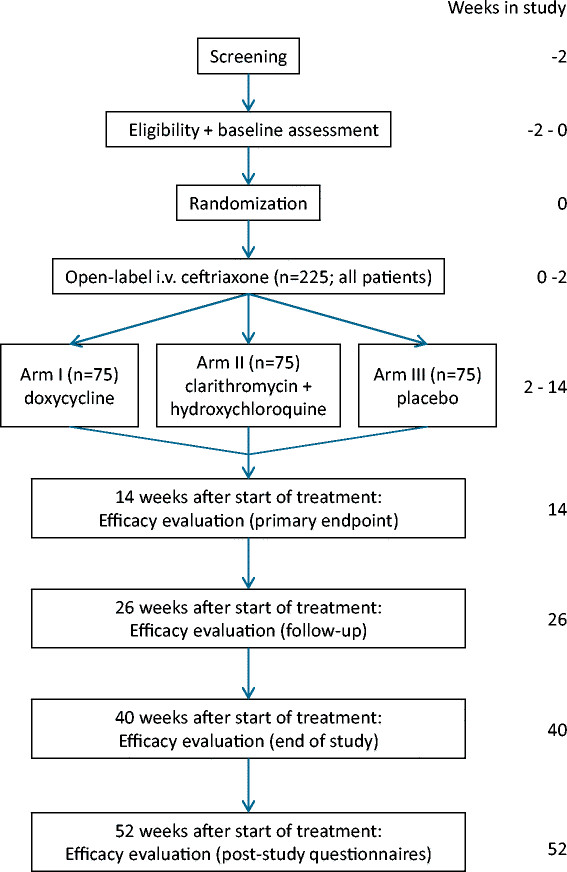
**Flowchart trial design.**

After obtaining informed consent and completing the baseline assessment, patients are randomly assigned to one of three groups in a 1:1:1 allocation ratio (Figure 1). The randomization is computerized and balanced by minimization for age (<or ≥40 years), gender, duration of symptoms (<or ≥1 year), and baseline Global Health Composite score of the RAND-36 Health Status Inventory (RAND SF-36), consisting of all RAND SF-36 subscales [22]. The randomization list consists of consecutive medication numbers that are entered into a secured web-based database by an independent web manager. All personnel involved in the study (except the web manager and study pharmacist) and participants are masked to treatment allocation. If the code is broken, it renders the patient non-eligible. To assess success of masking, patients are asked at the week 14 evaluation whether they think they have received oral antibiotics or placebo.

### Intervention

All patients are treated with open-label intravenous (i.v.) ceftriaxone 2000 mg qd via a peripheral i.v. catheter for 14 days. To monitor side effects, patients are admitted to the Sint Maartenskliniek for administration during day 1 and 2. Subsequent doses, prepared by the Sint Maartenskliniek Pharmacy, are given intravenously in a home-care setting by specialized nurses. After completion of ceftriaxone treatment, patients start with the randomized, blinded, oral study drugs. The oral drug regimen comprises either (I) doxycycline 100 mg b.i.d. combined with a placebo b.i.d. for 12 weeks, (II) clarithromycin 500 mg b.i.d. combined with hydroxychloroquine 200 mg b.i.d. for 12 weeks, or (III) double placebo b.i.d. for 12 weeks. The study drugs are to be taken twice daily after the meals. Study drugs and placebo are prepared as capsules with identical appearance. Preparation and labeling of doxycycline, clarithromycin, hydroxychloroquine, and placebo is performed by the Clinical Trials Unit of the Department of Clinical Pharmacy of the Radboudumc according to Good Manufacturing Practice (GMP) guidelines. Drug utilization is assessed by pill counting. Compliance is verified by using patient diaries and MEMS (Medication Event Monitoring System) caps [23],[24].

### Concomitant medication

Any antibacterial drugs other than study medications are prohibited during the entire study period. In case of proven intercurrent infections (e.g., urinary tract infection), specific antimicrobial therapy may be given for a maximum of 5 days. Indications should be discussed with the investigator, and efforts should be made to select an antimicrobial drug with no *in vitro* activity against *B. burgdorferi*. The following drugs are prohibited because of potential interaction with study drugs or potential effects on efficacy of treatment: cisapride, astemizole, terfenadine, barbiturates, phenytoin, carbamazepine, prednisone, recombinant cytokines, hematopoietic growth factors, or immunoglobulins. If treatment with one of these drugs is required, the patient will be classified as therapy discontinuation.

### Assessments

An extensive baseline assessment is performed including questionnaires, measurement of physical activity, and clinical, laboratory, microbiological, and neuropsychological evaluation. Neurological symptoms are assessed by the lead study physicians using a standardized interview and clinical neurological examination at baseline and week 14 [25].

Study visits for safety evaluation are performed at week 2, week 8, and week 14 after baseline. Safety assessments include a medical history, physical examination, and laboratory investigation (hemoglobin, hematocrit, leukocytes, platelets, glucose, creatinin, alkaline phosphatase, alanine aminotransferase).

Efficacy evaluation is performed at week 14 (end of treatment period, EOT), week 26 (12 weeks after EOT), and week 40 after baseline (end of study, EOS, 26 weeks after EOT). After the last comprehensive outcome assessment at week 40, patients are surveyed by post-study questionnaires at week 52.

### Outcome measures

The primary outcome measure is health-related quality of life at EOT (week 14), assessed by the physical component summary score (PCS) of the RAND-36 Health Status Inventory (RAND SF-36) [22]. This score is based on the weighed subscale scores of the four physical RAND SF-36 subscales (physical functioning, role limitations due to physical health problems, pain, and general health perceptions). The PCS is transformed to norm-based T-scores (with a mean of 50 and a standard deviation of 10 in the general population) and ranges from 15 to 61, with higher scores indicating a better physical quality of life.

Main secondary endpoints include:

- (a) Physical and mental aspects of health-related quality of life, assessed by the subscales of the RAND SF-36 (physical functioning, role limitations due to physical health problems, pain, general health perceptions, emotional well-being (also known as mental health), role limitations due to emotional problems, social functioning, and energy/fatigue (also known as vitality).
- (b) Fatigue, assessed by the Fatigue Severity subscale of the Checklist Individual Strength (CIS) [26]. The CIS is a reliable instrument with good validity and sensitivity to change in patients with rheumatoid arthritis, fibromyalgia, and chronic fatigue syndrome [26]-[28].
- (c) Neuropsychological assessment covering the five major cognitive domains, based on a similar test battery previously used to measure borreliosis-related impairment [7],[29],[30]. *Episodic memory* is assessed using the Rey Auditory Verbal Learning Test, using parallel versions for the follow-up assessments to reduce nonspecific learning effects [31]. *Attention*/*Working memory* is assessed using the Digit Span test [32]. *Language* is measured with the Category Fluency test (animal/profession naming) [33]. *Speed of information processing* is assessed using the Trail Making Test (TMT) part A [34], the average speed of Cards I and II from the Stroop Color-Word Test [35], and the Symbol-Digit Substitution Test [36]. *Executive functions* are measured using the TMT Interference score (Part B/Part A) and the Stroop interference score (Card III/average of Cards I and II) [37]. To identify participants who display suboptimal effort affecting symptom validity, the Amsterdam Short Term Memory Test is administered at baseline [38]. The entire test battery requires approximately 1 hour to be completed and is performed according to a standardized protocol by three psychologists, who have been trained in test administration and scoring.
- (d) Physical activity during 12 days, measured by an actometer. An actometer is a three-dimensional motion device (43*29*16 mm) with a piezoelectric sensor that is worn around the ankle [39]. Sensor signals are stored every five minutes, from which mean Daily Physical Activity scores are computed. Actometers have been shown to yield valid and highly reliable data [39],[40].

### Economic evaluation

To determine the cost-effectiveness of the different antibiotic regimens, an economic evaluation is conducted, and these results will be published separately. This cost-utility analysis investigates the potential efficiency of short-term antibiotic therapy (2 weeks) versus long-term antibiotic therapy (14 weeks) from a societal perspective. Primary outcome measures are costs and quality-adjusted life years (QALYs). For the overall quantification of health status as a single index, the Dutch version of the standard EQ-5D classification system developed by the EuroQol Group is used [41]. QALYs will be estimated from the EQ-5D scores over a one-year period using the trapezium method.

The cost analysis consists of two main parts. First, volumes of care are measured prospectively using a structured survey. Productivity losses for patients are estimated using the Short Form - Health and Labour Questionnaire (SF-HLQ) [42],[43]. The friction cost method will be applied [44],[45]. In the second part of the cost analysis, prices will be determined for each unit of care consumed using the Dutch manual for cost research [44]. The cost-effectiveness analysis will consist of computing the incremental cost effectiveness ratio (ICER) by dividing the mean difference in total costs by the mean difference in QALYs. Insight into parameter uncertainty will be obtained with the bootstrap method and will be presented as cost-effectiveness acceptability curves.

### Safety monitoring

Safety is evaluated by clinical laboratory tests and physical examinations. All observed and reported adverse events, regardless of suspected causal relationship, are recorded. An independent external data and safety monitoring board (DSMB) will review the blinded safety data after the first 60 patients have reached the end of treatment study visit. The DSMB may decide to recommend study termination or protocol modifications if required by the safety data or trial conduct.

### Statistical analysis

Data will be analyzed according to the modified intention-to-treat (mITT) principle. Patients who have been randomized into the study and received at least one dose of ceftriaxone are included in the mITT analysis group. In the primary analysis, analysis of covariance (ANCOVA) will be used to compare the three study arms, with gender and the baseline value of the dependent variable as covariates. Pairwise comparisons are performed for the different treatment modalities with Bonferroni correction for multiple comparisons. Missing data will be imputed by carrying the last observation forward, in order to obtain a conservative estimate of the treatment effect. No interim efficacy analysis will be performed. Two-sided 5% significance levels will be used to identify statistically significant results. All confidence intervals reported will be 95% confidence intervals. All statistical analyses will be performed using SPSS software.

A sensitivity analysis will be performed on the per-protocol subgroup. The per-protocol population comprises of patients for whom all of the following apply: has met the in- and exclusion criteria; has taken at least 75% of the study drugs as recorded by MEMS; has not taken any other antimicrobial drug for more than 5 days during the study period; has not taken any prohibited concomitant medication; has not been unblinded before end of study.

When a difference between one of the experimental treatments (ceftriaxone followed by 12 weeks of either of the oral treatments) and the reference treatment (two weeks ceftriaxone followed by placebo) is found, subgroup analyses will be performed to identify factors that may affect the treatment outcome. This will be done by adding the factors and their interaction with the treatment to the analysis of covariance model. The duration of treatment effect will be evaluated in an explorative way with linear mixed models. When an outcome variable is measured more than once, a random (patient-dependent) intercept will be included in the analysis.

To evaluate the neuropsychological outcomes, results on individual tests will be standardized into z-scores to make across-test comparison possible (using baseline group mean and standard deviation as reference), and averaged into cognitive domain scores. Higher z-scores reflect a better performance. If necessary, scales will be inverted, e.g., in the case of reaction times where higher scores reflect a slower performance.

### Sample size

The final power calculation was based on a pilot study on 80 patients with borreliosis-attributed persistent symptoms (Berende et al., unpublished). Patients were classified as having a poor or reasonable clinical condition as assessed during the first clinical consultation at the outpatient clinic. The difference in the PCS score between patients with a poor and those with a reasonable clinical condition was 3 points, with a standard deviation of 8. This corresponds with the minimally clinically important difference (MCID) of 2 to 5 points that has been proposed for the PCS [46]. In order to detect a difference of 3 points with a power of 90%, a two-sided alpha of 5% and a reliability coefficient (correlation between consecutive measurements) of 0.7 [47], a minimum of 75 patients are required per treatment group (225 patients in total). To compensate for possible loss to follow-up, a study population of at least 255 patients is targeted for.

## Discussion

The PLEASE study evaluates whether long-term antibiotic treatment of patients diagnosed with borreliosis-attributed persistent symptoms is efficient and leads to better patient outcome than short-term treatment. So far, there are few prospective, controlled data to support prolonged antibiotic treatment. Indeed, some studies have suggested positive outcomes on selected endpoints, such as persistent fatigue [17], cognitive functioning [29], quality of life [18], or clinical response rate [48], in specific groups of patients with putative persistent Lyme disease. However, these results were generally disappointing, and cannot be generalized. Other randomized clinical trials have not demonstrated beneficial effects of prolonged antibiotic treatment [10],[30]. Importantly, all of these studies were performed in North America. Borreliosis is caused by different *Borrelia* species in the US and Europe, with different clinical manifestations [49]. The present study will be the first randomized clinical trial to study long-term antibiotic treatment for borreliosis-attributed persistent symptoms in Europe.

The strategic choices leading to the design of a prospective, randomized, 3-arm study are complex. First, i.v. ceftriaxone followed by doxycycline is generally considered the gold standard therapy for complicated borreliosis [10]. Whereas administration of ceftriaxone for longer than 2 weeks has been advocated, a randomized, open-label study was unable to demonstrate that ceftriaxone treatment for 4 weeks would be significantly better [48].

Prolonged therapy with oral doxycycline has been associated with success in a large case series of patients with borreliosis-attributed persistent symptoms [50]. Data from another case series suggested that combined therapy with oral clarithromycin and hydroxychloroquine for at least 3 months may be at least as effective as prolonged doxycycline [51]. Hydroxychloroquine increases the lysosomal pH and is hypothesized to increase macrolide activity [52]. However, few conclusions can be drawn from those clinical studies, as they were retrospective, uncontrolled, observational studies. Based on these considerations, the present study was designed to compare a 12 weeks' course of doxycycline to 12 weeks of clarithromycin and hydroxychloroquine versus placebo.

To provide a standard treatment for all patients, and to cover potentially undiagnosed neuroborreliosis, all randomized patients receive an open-label course of i.v. ceftriaxone for 2 weeks preceding randomized blinded study drugs. In this respect, the present study differs from previous trials comparing prolonged therapy to placebo [10],[17],[18],[29]. By applying a standardized open-label treatment to patients in all treatment arms, the study is designed to compare short-term standard treatment [15] to prolonged therapy as advocated by several position papers [16],[53]. In addition, this approach does not leave potentially active infection untreated in patients who are randomized to the control arm, and it also controls for the wide variation in prior antibiotic therapies (or lack thereof) that patients with borreliosis-attributed persistent symptoms may have received.

As the primary outcome measure, we have chosen the physical component summary score (PCS) of the RAND-36 Health Status Inventory (RAND SF-36) [22]. The RAND SF-36 is similar to the Medical Outcomes Study (MOS) 36-item Short-Form General Health Survey (SF-36) [54]. The PCS, also known as the physical health composite score (PHC) [22], is computed by a non-orthogonal scoring algorithm. Several previous studies have used the alternate (SF-36) version of the PCS, applying a principal components analysis with orthogonal factors, with mental health components contributing negatively to this PCS score [54]. This SF-36 PCS has proven difficult to interpret as the level of mental health influences the physical health score and is therefore not purely a reflection of physical health. Furthermore, the SF-36 PCS is less sensitive to change than the underlying scales, while the RAND SF-36 PCS has been shown to be sensitive to change [55]-[62]. Despite the differences in calculation of both composite scores, they do correlate highly, indicating that they do represent similar constructs [58],[59].

In conclusion, the PLEASE study is expected to provide evidence for prescribing or withholding prolonged antibiotic treatment as compared to standard short-term treatment in patients with borreliosis-attributed persistent symptoms. In addition, this study may help to define subgroups of patients who may or may not benefit from additional antibiotic treatment, and contribute to a more cost-effective management of this disease entity.

## Authors’ original submitted files for images

Below are the links to the authors’ original submitted files for images. Authors’ original file for figure 1

## Abbreviations

PLEASE: Persistent Lyme Empiric Antibiotic Study Europe
i.v: intravenous
PCS: Physical component summary score
RAND SF-36: RAND-36 Health Status Inventory
EM: Erythema migrans
IDSA: Infectious Diseases Society of America
ILADS: International Lyme and Associated Diseases Society
GMP: Good Manufacturing Practice
MEMS: Medication Event Monitoring System
CIS: Checklist Individual Strength
TMT: Trail Making Test
QALY: Quality-adjusted life year
SF-HLQ: Short Form - Health and Labour Questionnaire
ICER: Incremental cost effectiveness ratio
DSMB: Data and safety monitoring board
mITT: modified intention-to-treat
ANCOVA: Analysis of covariance
MCID: Minimally clinically important difference
PCR: Polymerase chain reaction
CSF: cerebrospinal fluid
ALP: Alkaline phosfatase
ALT: Alanine aminotransferase
AST: Aspartate aminotransferase.
