# Supplementary figures and images for: Persistent Lyme Empiric Antibiotic Study Europe (PLEASE) - design of a randomized controlled trial of prolonged antibiotic treatment in patients with persistent symptoms attributed to Lyme borreliosis
Source: BMC Infect Dis. 2014 Oct 16;14:543. doi: 10.1186/s12879-014-0543-y (PMC4203907; doi:10.1186/s12879-014-0543-y)

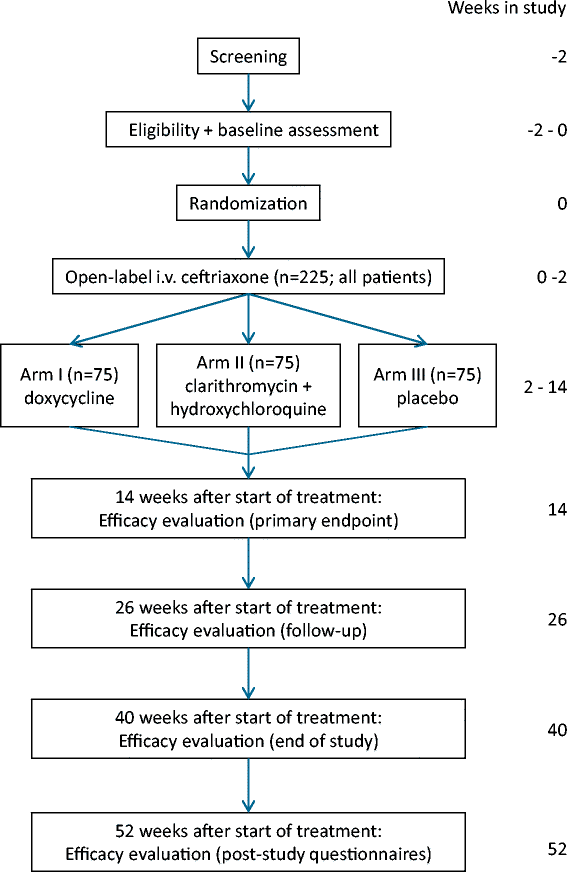

Supplement: Supplementary file 1 — Authors’ original file for figure 1 [file 12879_2014_543_MOESM1_ESM.gif]
